# Transcriptome analysis provides insights into the root response of Chinese fir to phosphorus deficiency

**DOI:** 10.1186/s12870-021-03245-6

**Published:** 2021-11-10

**Authors:** Wanting Chen, Mengyan Zhou, Mingzhen Zhao, Ranhong Chen, Mulualem Tigabu, Pengfei Wu, Ming Li, Xiangqing Ma

**Affiliations:** 1 Forestry College, Fujian Agriculture and Forestry University, Fuzhou, 350002 Fujian China; 2 Chinese Fir Engineering and Technological Research Center, National Forestry and Grassland Administration, Fuzhou, 350002 Fujian China; 3 Southern Swedish Forest Research Center, Faculty of Forest Science, Swedish University of Agricultural Sciences, PO Box 49, Alnarp, SE-230 53 Uppsala, Sweden

**Keywords:** *Cunninghamia lanceolata*, Phosphorus deficiency, Transcriptome, Differential expression genes, Citric acid and glyoxylate cycle pathway

## Abstract

**Background:**

Phosphorus is one of the essential elements for plant growth and development, but available phosphorus (Pi) content in many soil types is low. As a fast-growing tree species for timber production, Chinese fir is in great demand of Pi, and the lack of Pi in soil restricts the increase of productivity of Chinese fir plantation. Root morphology and the synthesis and secretion of organic acids play an important role in the uptake of phosphorus, but the molecular mechanisms of Chinese fir root responses to Pi deficiency are largely unexplored. In this study, seedlings of Yang 061 clone were grown under three Pi supply levels (0, 5 and 10 mg·L-1 P) and morphological attributes, organic acid content and enzyme activity were measured. The transcriptome data of Chinese fir root system were obtained and the expression levels of phosphorus responsive genes and organic acid synthesis related genes on citric acid and glyoxylate cycle pathway were determined.

**Results:**

We annotated 50,808 Unigenes from the transcriptome of Chinese fir roots. Among differentially expressed genes, seven genes of phosphate transporter family and 17 genes of purple acid phosphatase family were up-regulated by Pi deficiency, two proteins of SPX domain were up-regulated and one was down-regulated. The metabolic pathways of the citric acid and glyoxylate cycle pathway were mapped, and the expression characteristics of the related Unigenes under different phosphorus treatments were analyzed. The genes involved in malic acid and citric acid synthesis were up-regulated, and the activities of the related enzymes were significantly enhanced under long-term Pi stress. The contents of citric acid and malic acid in the roots of Chinese fir increased after 30 days of Pi deficiency.

**Conclusion:**

Chinese fir roots showed increased expression of genes related with phosphorus starvation, citrate and malate synthesis genes, increased content of organic acids, and enhanced activities of related enzymes under Pi deficiency. The results provide a new insight for revealing the molecular mechanism of adaption to Pi deficiency and the pathway of organic acid synthesis in Chinese fir roots.

## Background

Phosphorus migrates slowly in the soil and moves over a short distance [22]. The inorganic phosphate ions are often fixed by the abundant calcium, iron, aluminum and clay in the soil to form insoluble phosphate precipitates such as Ca-P, Fe-P, Al-P and O-P. Thus, more than 95% of soil available phosphorus (Pi) cannot be directly absorbed and utilized by plants, resulting in low plant available phosphorus [30, 42, 53]. Acidic red soil and latosol in southern China are highly aluminized, and the solubility of aluminum and iron in strong acidic solutions is high. This makes it easier to combine with phosphorus to form insoluble phosphate precipitates, which results in very low content of available phosphorus in the soil for direct absorption and utilization by plants [7, 24, 63].

Chinese fir (*Cunninghamia lanceolata*) is the largest timber tree species widely grown in Southern China [5, 62]. Due to strong chemical fixation of phosphorus in red soil, Chinese fir plantations are often faced with severe Pi deficiency, which limits its growth and yield [9]. Furthermore, the long-term pure forest multi-generation continuous planting management mode aggravates availability of soil nutrients, particularly available phosphorus. Thus, the deficiency of plant available phosphorus in soils where Chinese fir is growing has become an important limiting factor for the improvement of yield and quality of Chinese fir plantation [41, 60].

Plants adapt to soil P deficiency through various strategies, including changes in root quantity (e.g. biomass and length) and morphology (e.g. specific root area and specific root length) for efficiently explore the soil, as well as altering root exudates and mycorrhizal symbiosis to increase P bioavailability in soils [2, 6, 21, 31, 54, 58]. The gene regulatory network of plant response to Pi deficiency is complex, and there are differences among different plant species. The growth and development of plant roots are very sensitive to nutrients, and the lateral roots usually propagate preferentially in nutrient-rich areas to adapt to the uneven distribution of nutrients [40]. When the content of phosphorus availability is low, the plants will increase the distribution of carbon sources, promote the growth of lateral roots, increase the number and length of root hairs, increase the distribution of root system in shallow soil, and increase the contact surface area between root system and soil, forming an “umbrella-like” root configuration [12, 14, 33, 49, 69]. The effects of Pi deficiency on root morphology were controlled by the expression of auxin, cytokinin, gibberellin and a series of related genes [8, 39, 47, 65, 67]. However, few candidate genes are related to the change of root structure under Pi deficiency condition. In addition, organic anions from root exudates are also considered important factors in mobilizing soil phosphorus and promoting phosphorus uptake. Exogenous organophosphates are activated by root exudates, Pi deficiency signals are transmitted by the distribution of photoassimilates, and adaptive capacity is enhanced by increased activity of acid phosphatase in the root system [4, 8, 19, 25, 64]. However, the study on molecular mechanism of organic acid biosynthesis and efflux under Pi deficiency condition is limited. Studies on model plants, such as Arabidopsis and rice, showed that Pi deficiency induces the gene expression of key enzymes related to organic acid synthesis, such as malate dehydrogenase (MDH) and citrate synthase (CS), and the related protein synthesis also accumulates significantly to promote the synthesis of organic acids in plant roots [48, 51, 59].

Advances in molecular biology in recent decades have revealed the central role of phosphate starvation genes (PHR1) in the transcription response associated with Pi deficiency [26, 36, 38]. PHR1, the key transcription factor, has also been found in Chinese fir [43, 61]. *ClPHR1*was expressed in all tissues of Chinese fir, but the highest expression level is found in the root. Eight differentially expressed genes related to phosphorus were found in the transcriptome of Chinese fir root under aluminum stress, which indicated that the transportation and metabolism of phosphate in Chinese fir root system are also affected [28]. The relationship between the transcriptional regulation and phosphorus assimilation in the roots of slow-growing and fast-growing Chinese fir was also studied [13]. It was found that the acid phosphatase (ACP) and phosphoenolpyruvate carboxylase (PEPC) activities in the roots of slow-growing Chinese fir were higher, and the phosphorus concentration in the roots was higher in slow-growing provenances and more efficient in phosphorus uptake and translocation than fast-growing provenances, while fast-growing provenances were more efficient than slow-growing provenances in biomass production. At the same time, phosphorus deficiency resulted in overexpression of genes involved in Pi acquisition and transport including phosphate transporter gene *PHT1;4* and *PHO1*, *ACP*, and *MDH* in Chinese fir, while high Pi decreased *PHT1;4* transcription and increased mRNA level of phosphate transporter *PHT2;1* gene. These findings lay a foundation to study further the molecular mechanism of phosphorus deficiency in Chinese fir root system and related genes and metabolites. As the molecular mechanism of morphological and physiological responses of Chinese fir to Pi deficiency is not clear, further studies on the expression and regulation of genes induced by Pi deficiency need to be carried out.

In this study, the transcriptional characteristics of Chinese fir roots under Pi deficiency were studied by measuring the root transcriptome of the seedlings of Yang 061 Chinese fir clone, which were treated with no phosphorus, low phosphorus and normal phosphorus supply for 3 and 30 days. The differentially expressed genes and the related metabolic pathways under different phosphorus supply conditions were analyzed to screen the candidate genes that might play a role in Chinese fir adaptation to Pi deficiency. Identification and analysis of the functional expression of key genes involved in phosphorus transport, acid phosphatase secretion, and organic acid metabolism, in combination with the determination of some metabolites in the metabolic pathway of significantly different gene enrichment were studied. The results of this study are of great significance in revealing the transcriptional characteristics and molecular mechanism of Chinese fir roots in response to Pi deficiency, and providing gene resources and data support for screening and breeding Chinese fir genotypes for high phosphorus utilization.

## Methods

### Treatments and growth conditions

The experiment was carried out in greenhouse in Forestry College (26°04′N, 119°14′E), Fujian Agriculture and Forestry University. The region has an altitude of 10 m above sea level and a subtropical monsoon climate, with an average annual temperature of 25 °C, an average annual sunshine of 1700–1980 h and an average annual precipitation of 900–2100 mm. The experimental material, one-year-old seedlings of Yang 061 clone of Chinese fir provided by the state-owned forest farm of Yangkou, Fujian, China, were cultured with 1/3 Hoagland nutrient solution [56] in sand media for 30 days in the greenhouse at Fujian Agriculture and Forestry University, China. Then the following treatments were applied to the seedlings with similar growth vigor: phosphorus starvation (NP, 0 mmol·L^− 1^ KH_2_PO_4_ and 0.333 mmol·L^− 1^KCl; 0 mg·L^− 1^ P), low phosphorus supply (LP, 0.167 mmol·L^− 1^ KH_2_PO_4_ and 0.167 mmol·L^− 1^KCl; 5 mg·L^− 1^ P) and high phosphorus supply treatments (HP, 0.333 mmol·L^− 1^ KH_2_PO_4_; 10 mg·L^− 1^ P). After 3 and 30 days of growth under treatment conditions, root samples were collected from NP-, LP- and HP-treated plants, organic acids and related enzymes were extracted, and the transcriptome and gene expression of the roots were analyzed. Seedling height, root morphology, biomass and nutrient content were also determined after 30 and 60 days of growth.

For root morphology determination, whole root systems of intact plants were carefully uprooted and washed with deionized water to remove the nutrient solution. Root length and root surface area were measured using WinRHIZO (Epson 1680, WinRHIZO Pro2003, Regent Instruments Inc., Quebec, Canada). The dry weight (DW) of shoots and roots were measured respectively after oven-drying for 45 min at 105 °C and 48 h at 65 °C until constant mass. Nutrient element concentrations were subsequently determined by inductively coupled plasma atomic emission spectroscopy.

### RNA extraction and quality control

The roots of plants treated with NP-, LP- and HP- supply for 3 and 30 days were harvested for RNA-Seq with three independent biological replicates for each treatment. Total RNA was extracted using a Spectrum™ Plant Total RNA Kit (Sigma-Aldrich, St Louis, MO, USA) and RNA quantity and quality were assessed by a NanoDrop spectrophotometer (NanoDrop Technologies, Wilmington, DE, USA) and Agilent 2100 Bioanalyzer (Agilent Technologies, Santa Clara, CA, USA). The mRNA was enriched by magnetic beads with Oligo (dT) for RNASeq. The constructed library was sequenced with Illumina HiSeqTM4000 (Illumina, San Diego, CA, USA) at GENEDENOVO (www.genedenovo.com/).

### Sequence processing and analysis

Raw reads were quality checked, trimmed and de novo assembled using Trinity 5. Gene expression was calculated and normalized using reads per kb per million reads (RPKM) [32]. Differential expression between the Pi deficiency treatment groups was analyzed by edger (DOI: 10.18129/B9.bioc.edgeR) and up−/down- regulation of genes was considered to be significant if its expression was ≥2-fold (*P* < 0.05). The filtered transcripts were selected to be denovo Chinese fir root transcriptome, used as a reference for further analysis. The denovo Chinese fir root transcriptome sequences were a combination of the differentially expressed transcripts and other transcripts with RPKM ≥1.5 regardless of P treatments. These sequences were annotated using Blast2Go [16] and MapMan [45].

### Real-time quantitative reverse transcription PCR analysis

The first-strand cDNA was synthesized from 1.0 μg RNA using GoScriptTM Reverse Transcription System (Promega, Madison, Wisconsin, America). The reverse transcription system was based on 2 μg RNA in a final volume of 20 μL reaction volume. Reverse transcription was performed at 42 °C for 60 min with a final denaturation at 70 °C for 5 min. The resulting cDNA was diluted to100 ng/μL with nuclease-free water and stored at − 20 °C.

The qRT-PCR reactions were carried out on a StepOnePlus™ real-time fluorescence quantitative PCR instrument (Corbett Research, AUS) using GoTag qPCR Master Mix (Promega, USA) with transcript-specific primers. The qPCR was performed on a mixture containing 10 μL 2× SYBR Premix Ex Taq™, 0.5 uL each of specific forward and reverse primers and 2 uL cDNA. The amplification program was 95 °C for 3 min followed by 40 cycles of 95 °C for 20 s, 60 °C for 30 s, and 72 °C for 30 s. The specificity of the amplification was controlled by melting curve analysis (from 65 to 95 °C). We used β-Actin1(Forward: 5′- CTCTCTCAGCACCTTCGAGCAG − 3′, Reverse: 5′- TCCACATACAACCGCTCCACTG − 3′) as a reference gene to normalize expression level [10]. The relative expression of genes in different Pi treatments over time was calculated by 2^−ΔΔCt^ method. Three biological replicates of each treatment and three technical replicates of each sample were used in the analysis.

### Organic acids and related enzymes extraction

The roots of plants treated with NP-, LP- and HP- supply for 3,10,30 days were used for organic acids and related enzymes extraction. Root samples (0.2 g) were added into 1 ml extraction solution, ultrasonically extracted for 60 min, and the supernatant was filtered with needle-type filter and analyzed using HPLC. The HPLC conditions were Agilent 1100 High-performance liquid chromatography (Agilent, USA), Kromasil C18(AKZO NOBEL, SE) reversed phase column (250 mm × 4.6 mm, 5 μm), 10 μL injection volume, 0.8 mL / min flow rate, 25 °C column temperature, 20 min sampling time and 214 nm UV wavelength. The contents of malic acid (standard curve: y = 0.4409x + 0.2274; R^2^ = 0.9992) and citric acid (standard curve: y = 0.1673x + 0.5810; R^2^ = 0.9990) were determined. The injected sample was 10 μL, the flow rate was 1.0 mL / min, the column temperature was 25 °C, the retention time was 40 min and the wavelength was 325 nm. The pyruvic acid (standard curve: y = 12.033x-2.4087; R^2^ = 0.9992) content was also determined. The citrate synthase (CS), NAD-malate dehydrogenase (NAD-MDH), pyruvate dehydrogenase (PDH), phosphoenolpyruvate carboxylase (PEPC) Kits (Solarbio, CN) were used to determine the activity of organic acid related enzymes involved in phosphorus metabolism.

## Results

### Plant growth and root morphology

**Fig. 1:**
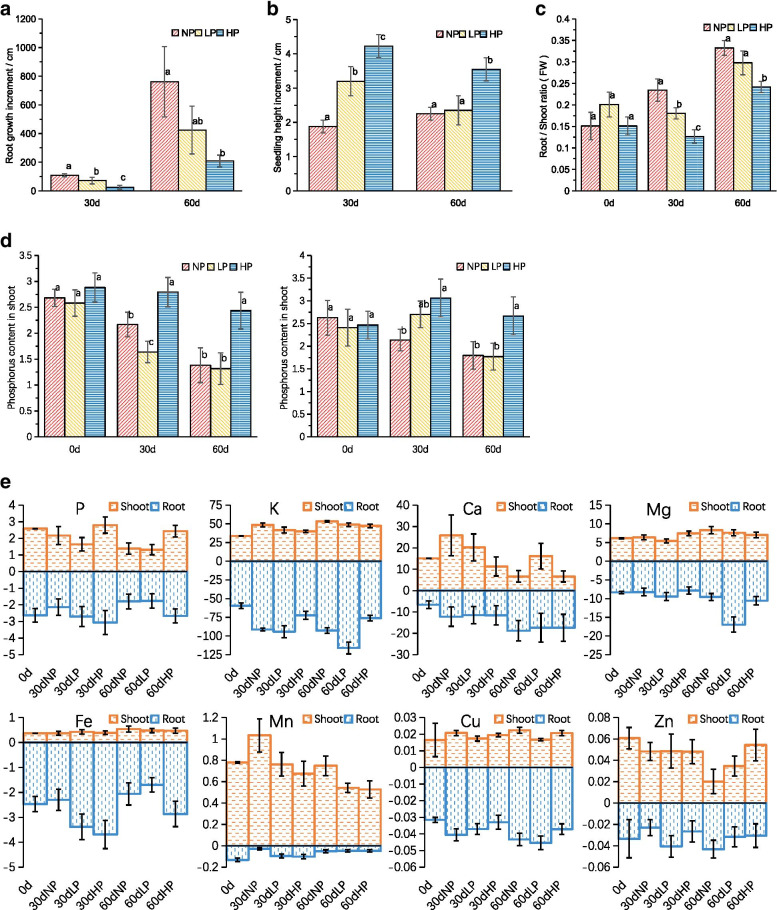
Morphological and physiological responses of Chinese fir seedlings cultured for 30 and 60 days under different phosphorus conditions. **A** Height of Chinese fir seedlings; **B** Root growth of Chinese fir seedlings; **C** Root/shoot ratio of Chinese fir seedlings; **D** Phosphorus content in shoot and root of Chinese fir seedlings; **E** Element contents respond to Pi starvation. Values are means ± SD (*n* = 3). Bars followed by different letter(s) indicate significant difference at *P* < 0.01

After 30 and 60 days of growth under the experimental conditions, the height growth of Chinese fir seedlings under Pi deficiency condition was significantly lower than that of Chinese fir seedlings grown under high Pi supply (Fig. 1A). On the contrary, the root growth of Chinese fir under Pi deficiency condition was significantly higher than that under normal Pi supply condition (Fig. 1B). The root/shoot ratio of Chinese fir seedlings under Pi deficiency was higher than that under normal condition (Fig.1C). As a whole, Pi deficiency inhibited seedling height growth and promoted root elongation compared with Pi sufficient conditions.

After 30 and 60 days of growth under different treatment conditions, phosphorus content in roots decreased significantly in Pi-deficient seedlings compared with Pi-sufficient seedlings (Fig. 1D). The phosphorus concentrations in the shoot and root of plants grown without phosphorus at 30 days of stress were only 78 and 70% of those grown under adequate phosphorus condition, respectively, and further decreased to 57 and 67% at 60 days.

Analysis of other nutrient elements revealed that Pi deficiency inhibited the accumulation of Mg in shoot, increased the accumulation of Ca and Mn, but no significant change in the accumulation of K, Mg, Cu and Zn (Fig. 1E). In Pi deficiency treatment, the changes of element contents in roots were different from those in shoot. Pi deficiency increased the concentrations of K and Cu in roots, decreased the concentrations of Fe, and the contents of Ca, Mg and Mn had no significant difference. Under Pi deficiency, the ratios of root/shoot element concentrations of P, K, Mg and Cu increased while that of Ca, Fe and Mn decreased.

### Functional annotation and classification

**Fig. 2:**
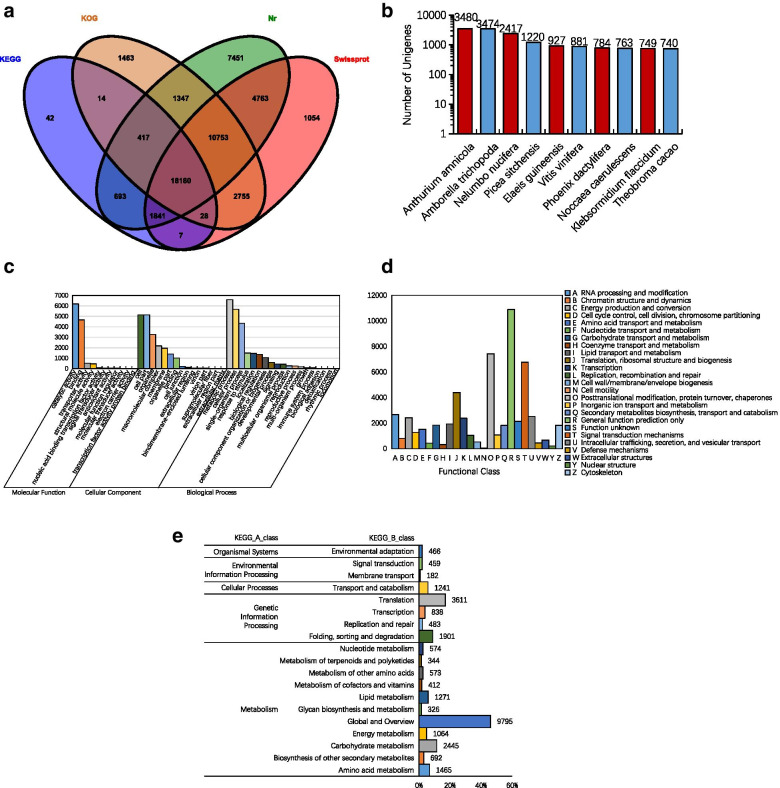
Functional annotation and classification of Unigenes using different databases. **A** Venn diagram of Unigenes annotated in the Nr, SwissProt, KOG and KEGG database; **B** Nr Database annotated statistical chart of homologous species where Y-axis indicates the number of Nr annotated genes and X-axis indicates the homologous species; **C** GO Functional classification annotation where Y-axis indicates the number of GO annotated genes and X-axis indicates the GO category; **D** KOG function annotation where Y-axis indicates the number of KOG annotated genes and X-axis indicates the KOG category classification of Unigenes in Chinese fir transcriptome; **E** KEGG annotated secondary classification results of Unigenes in Chinese fir transcriptome where Y-axis indicates the KEGG category and X-axis indicates the percentage of transcripts in KEGG annotated genes

We sequenced 18 samples by RNA-Seq technology and transcriptome sequencing data were deposited in the NCBI SRA database under the accession number PRJNA681878. We generated 126,413 unigenes with a maximum length of 16,707 bp, a minimum length of 201 bp, and a mean contig length of 702 bp. The assembled Unigene sequences were compared with the Nr, SwissProt, KOG and KEGG databases using blastx (Fig. 2A). A total of 50,808 Unigenes were annotated, and 45,445, 39,381, 34,957 and 21,222 Unigenes were annotated out of the four databases, accounting for 35.95, 31.15, 27.65 and 16.79% of total Unigenes respectively. Using blastx and Nr databases to do species distribution statistics (Fig. 2B), the results showed that, there were high homology between Chinese fir and *Anthurium amnicola* (7.66%), *Amborella trichopoda* (7.64%), *Nelumbo nucifera* (5.32%), *Picea sitchensis* (2.68%) and *Elaeis guineensis* (2.04%).

According to the Nr annotation information, the Gene Ontology (GO) annotation information of Unigenes was obtained by Blast2go, and all Unigenes were classified by WEGO, and the functional distribution characteristics of Unigenes were obtained (Fig. 2C). The most represented GO subcategories within the biological process main term were ‘cellular and metabolic processes’ and ‘cellular process’. The cellular component main term was mainly represented by the term ‘cell or cell part’ and ‘membrane or membrane part’. As for molecular function main term, 6196 transcripts corresponded to ‘catalytic activity’ and 4655 sequences to ‘binding category’. We used KOG to further classify gene sequences into homology (Fig. 2D). The KOG database contained 34,957 annotation results, grouped into 25 different biological functions. The ‘General function prediction only’ term contained the largest number of annotated Unigenes (10,894, or 18.96%), followed by ‘Posttranslational modification, protein turnover, chaperones’ term (7431, or 12.93%), and ‘Signal transduction mechanisms’ term (6762, or 11.77%).

In the KEGG annotated results (Fig. 2E), 21,222 Unigenes were annotated into 135 different metabolic pathways in five main categories and 19 subcategories. Unigenes were most involved in ‘Metabolic pathways’ (4221 Unigenes), accounting for 19.89% of KEGG annotated Unigenes, and 2246 Unigenes (10.58%) were annotated into ‘Biosynthesis of secondary metabolites’ term. Sucrose signaling, hormone synthesis, organic acid metabolism and phosphorus transport induced by Pi deficiency were closely related to starch and sucrose metabolism, plant hormone signal transduction, tricarboxylic acid cycle (TCA cycle) and ABC transporter in KEGG metabolic pathway, and 369, 307, 228 and 182 Unigenes were involved in these terms, respectively.

### Screening of differentially expressed genes

**Fig. 3:**
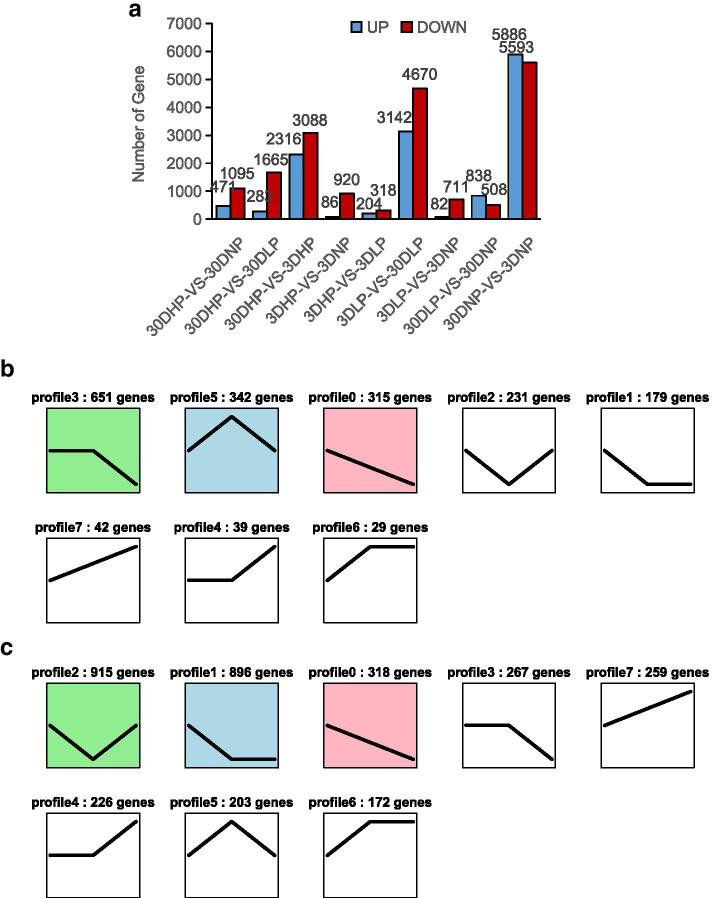
**A** Statistical histogram of differentially expressed genes between groups; **B** Trend gene numbers with different trend characteristics in 3-day HP-LP-NP subgroup; **C** Trend gene numbers with different trend characteristics in 30-day HP-LP-NP subgroup

Differentially expressed genes that were significant were analyzed among subgroups (Fig. 3A). After 3 days of stress treatment, 204 differentially expressed genes were up-regulated and 318 differentially expressed genes were down-regulated in low Pi group compared with normal Pi supply group. Compared with high Pi group, 86 differentially expressed genes were up-regulated and 920 differentially expressed genes were down-regulated in Pi starvation group while 82 differentially expressed genes up-regulated and 711 differentially expressed genes down-regulated compared with low Pi group. After 30 days of Pi deficiency treatment, 283 differentially expressed genes were up-regulated and 1665 differentially expressed genes were down-regulated in low Pi group compared with the normal Pi supply group, and 471 differentially expressed genes were up-regulated in Pi deficiency group compared with normal phosphorus supply group. There were 1095 differentially expressed genes (838 up-regulated and 508 down-regulated) in Pi starvation group and low Pi group.

From different subgroups, the number of down-regulated genes was more than up-regulated genes under Pi deficiency. Compared with HP treatment, HP versus NP subgroup had more differentially expressed genes than HP versus LP subgroup under the same treatment time. The number of differentially expressed genes in 30-day treatment was higher than that in 3-day treatment. The results of gene ontology categories with significantly differentially expressed gene among groups showed that the root system responded to Pi deficiency by changing its cell components, regulating its catalytic activity and adapting to the stress environment through various metabolic pathways. The KEGG pathway annotation of differentially expressed genes under Pi deficiency showed that the accumulation of oxidative phosphorylation, transmembrane transporters, osmotic stress response, hormone signal transduction, pyruvate metabolism, tricarboxylic acid cycle, acid phosphatase synthesis and other metabolic pathways were closely related to the responses of Chinese fir to Pi deficiency.

The gene expression patterns were clustered according to the characteristics of three consecutive samples, and the genes with certain biological characteristics were selected from the results of the clustering. The trends of differentially expressed genes in the 3-day HP-LP-NP subgroup and 30-day HP-LP-NP subgroup were analyzed. There were 1829 tendency genes in 3-day HP-LP-NP subgroup (Fig. 3B). Among them, 42 genes showed an upward trend of gene expression with the increase of Pi deficiency (Fig. 3B, profile7), and four Unigenes obtained annotation in GO and KEGG. The results showed that these four up-regulated genes were mainly involved in the phosphorylated carbohydrate metabolism of Chinese fir roots. There were 315 genes (Fig. 3B, profile0) that decreased in expression with increasing Pi deficiency. The GO annotation results were distributed in 20 subcategories. In the molecular function category, ‘catalytic activity’ term and ‘binding’ term enriched the most genes, and in the biological process category, ‘cellular’ process and ‘metabolic’ process accounted for the most. The top five pathways for KEGG annotation were microbial metabolism, lysine degradation, tricarboxylic acid (TCA) cycle, carbon metabolism and ribosome in different environments. There were 3257 tendency genes in 30-day HP-LP-NP subgroup (Fig. 3C). Among them, the expression of 259 genes increased with increasing Pi deficiency (Fig. 3C, profile7). There were 32 genes that got the GO annotation, participating in ‘metabolic process’ term, ‘cellular process’ term, ‘cell’ term, ‘cell part’ term, ‘catalytic activity’ term and ‘binding term’ respectively. KEGG annotated 33 genes of ascending tendency, and the pathway that enriched the most genes was biosynthesis and metabolic pathway of secondary metabolites. The trend analysis showed that there were 318 genes with the characteristic of decreasing expression with increased degree of Pi deficiency (Fig. 3C, profile0). There were 36 genes involved in the ‘metabolic process’, ‘cellular’ term, ‘cell’ term, ‘cell part’ term, ‘membrane’ term, ‘catalytic activity’ term. KEGG annotated 36 genes, including lipoic acid metabolism, phosphatidylinositol metabolism, starch and sucrose metabolism, tricarboxylic acid cycle, RNA degradation, protein export, RNA transport and other metabolic pathways.

### Genes putatively involved in pi deficiency response in Chinese fir root

**Fig. 4:**
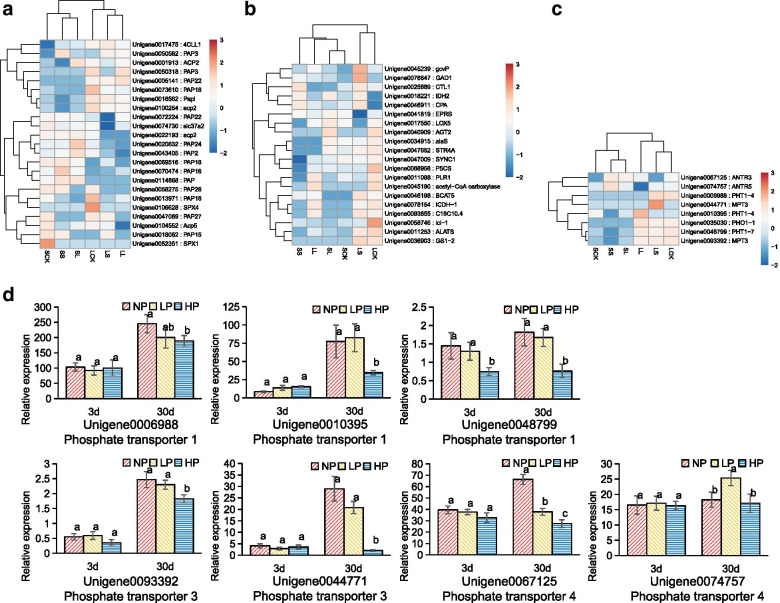
**A** Expression thermography of PAP, ACP and SPX genes in phosphorus response genes; **B** Thermograms of key genes for organic acid synthesis and metabolism; **C** Thermograms of PHT gene expression. The color bar indicates the expression levels [represented as log2(RPKM means)]; red indicates high expression level, blue indicates low expression level, and white indicates RPKM = 0; **D** Expression analysis of differentially expressed genes in PHTs gene family of the Chinese fir roots under different phosphorus concentrations (high Pi supply - HP, Pi deficiency - LP and Pi starvation -NP) for 3 and 30 days. The experiments were repeated three times on three biological replicates of each treatment (*n* = 3). Values are means ± SD. Bars followed by different letter(s) indicate significant difference at *P* < 0.01

When plants grow in phosphorus deficient conditions, the roots usually activate insoluble phosphate in the soil by secreting organic acids, and utilize the organic phosphorus around the rhizosphere by secreting acid phosphatase (ACP). Purple acid phosphatase (PAP) is a special class of acid phosphatase, and its members have different responses to Pi deficiency and their functions are regulated by transcription factors and SPX protein. SPX is an important regulator of phosphorus signaling network, which can interact with PHR1 to regulate the expression of phosphate transporter gene PHT1, thus promoting phosphorus uptake by roots under Pi deficiency. A total of 18 ACP genes, 70 PAP genes, 10 SPX structure and 10 protein genes were screened from the transcriptome data based on all Unigenes annotation in GO and KEGG databases. Among them, 17 PAP genes, three ACP genes and three SPX domain proteins were differentially expressed under Pi deficiency. The differentially expressed PAP, ACP, and SPX genes were analyzed by expression thermography (Fig. 4A), and the results showed that the expression of Unigene074730 in SPX gene increased and the expression of Unigene074730 in SPX gene decreased. Except ACP gene Unigene0100264 and PAP gene Unigene0013971 and Unigene104552, the expression of other ACP and PAP genes increased with the increase of Pi deficiency degree and the extension of stress time. The results showed that Chinese fir had the physiological response of synthesizing ACP to activate the organic phosphorus in its rhizosphere under Pi deficiency.

According to the annotation of GO and KEGG database, 20 differentially expressed genes related to organic acid synthesis and metabolism were screened. The gene expression of these 20 genes under different Pi deficiency was analyzed by thermography (Fig. 4B), and the results showed that the differentially expressed genes in the process of organic acid synthesis and metabolism of Chinese fir under Pi deficiency were up-regulated upon 30 days of exposure to Pi deficiency. However, the up-regulation of genes related to organic acid synthesis and metabolism was not obvious under 3-day Pi deficiency treatment.

Based on all Unigene annotation results, a total of 86 Unigene entries annotated as phosphate transporter family and phosphate protein carrier were retrieved. A total of 35 Unigene entries were confirmed to be involved in inorganic phosphate transport by sequence signature analysis compared to BLAST analysis. The other 51 genes may be involved in phosphorus transport, the function of which is unknown. Among the 35 phosphorus transporter genes, six genes, which had high homology with PHO1, were annotated as PHO1 family genes by Nr and Swissprot and classified as small molecule transporter by KOG. We found 16 genes with high homology with PHT1 family, inorganic phosphorus transporters; one carrier family with high homology to the PHT2 gene, sodium-dependent phosphate transporter; 12 carrier proteins annotated as mitochondrial phosphate, which were highly homologous and predicted to be PHT3 family. Based on the analysis of the characteristics of the PHT family genes, and the prediction of the coding sequence (CDS) and the characteristics of the PHT gene multiple sequence alignment with *Arabidopsis thaliana*, rice and poplar, four genes with high homology to PHT4 gene were screened. These genes were highly similar to the known PHT family genes. The Unigene annotation messages contained three probably anion transporters, whose pathway were annotated as perfuse of the major facilitator superfamily and one possibly organic anion transporter (Unigene 0074757). Furthermore, two PHT5 genes were annotated with membrane proteins containing SPX domains. A total of 41 genes involved in inorganic phosphate transport were obtained.

Thermographic analysis of seven Unigenes with significant differential expression of the PHT gene family (Fig. 4C) showed that Unigene0035030 (PREDICTED: PHO) was overexpressed after 30 days of growth in Pi deficient condition, but the expression level decreased with increasing the degree of Pi deficiency. Unigene0006988 (PREDICTED: PHT1) was enhanced after 30 days of Pi deficiency. The expression of Unigene0010395 (PREDICTED: PHT1) increased with increasing Pi deficiency time, and decreased with increasing the degree of Pi deficiency. The expressions of Unigene0048799 (PREDICTED: PHT1) and Unigene0093392 (PREDICTED: PHT3) increased with increasing Pi deficiency time while the expression of Unigene0044771 (PREDICTED: PHT3) was enhanced after 30 days of Pi deficiency. The expression of Unigene0067125 (PREDICTED: PHT4) and Unigene0074757 (PREDICTED: PHT4) increased with the decrease of phosphorus supply, the expression level was higher after 3 days than after 30 days.

In order to better understand the role of PHT gene in phosphate uptake by Chinese fir root, and clarify the expression pattern of PHT gene in Chinese fir root under different phosphorus supply treatments, seven known up-regulated PHT gene families expressed in phosphate starvation response were selected and verified by qRT-PCR (Fig. 4D) and fluorescence quantitative analysis. The expression of Unigene0006988 (PREDICTED: PHT1) increased with the increase of stress time, and significantly increased under the condition of Pi deficiency for 30 days. The expression of Unigene0010395 (PREDICTED: PHT1) increased with the increase of Pi stress time, but the expression of Pi starvation group was lower than low Pi group at the same P deficiency time. The expression of Unigene0048799 (PREDICTED: PHT1) increased with the increase of Pi deficiency time, and was significantly higher after 30 days than after 3 days. At the same time, the higher the stress degree, the higher the expression level, which was different from the result of gene expression heat map obtained by transcriptome sequencing. The expression level of Unigene0093392 (PREDICTED: PHT3) in different phosphorus supply groups was not significantly different after 3 days, but increased with the time of stress. The expression in high Pi treatment group was lower than in low Pi and Pi starvation groups after 30-day of growth. The expression of Unigene0044771 (PREDICTED: PHT3) increased with increasing Pi deficiency time and increased under Pi deficient condition after 30 days. The expression of Unigene0067125 (PREDICTED: PHT4) increased with increasing Pi deficiency time, and increased significantly after 30-day treatment. The expression level of Unigene0074757 (PREDICTED: PHT4) was higher under Pi deficient condition than that of normal phosphate treatment, and the expression level of Unigene0074757 increased noticeably after 30 days of Pi deficiency. The expression pattern of the rest of the genes was similar to that of RNA-seq.

### Citric acid and glyoxylate cycle pathway analysis associated with root-released organic acids

**Fig. 5:**
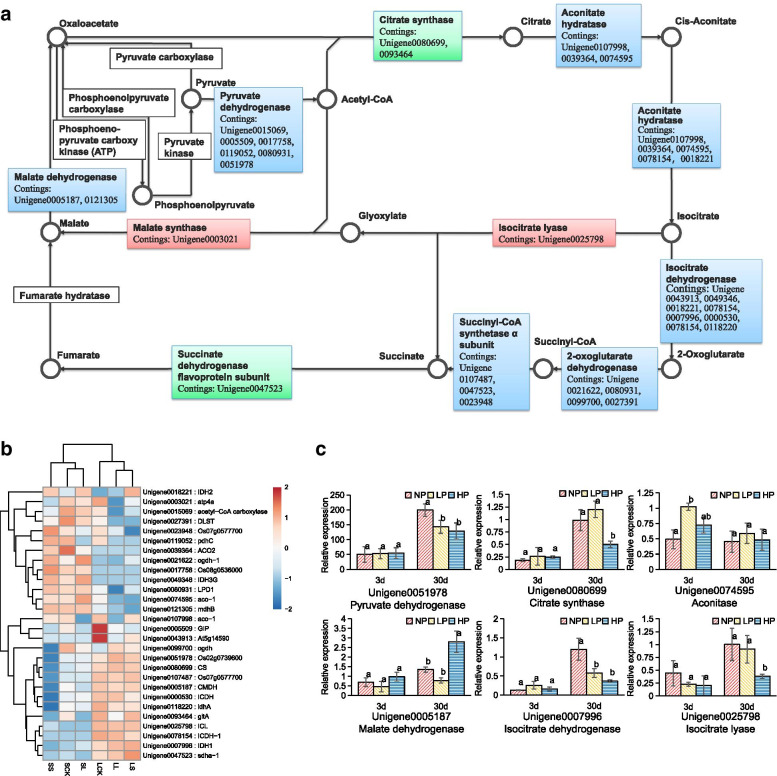
**A** Citric acid and glyoxylate cycle pathway. Red indicates the annotated genes with significant differences up-regulated under Pi deficiency, green indicates down-regulated expression, and blue represents the coexistence of up- and down-regulated annotated genes; **B** Thermograms of citric acid and glyoxylate cycle pathway related Unigene under different phosphorus treatments. The color bar indicates the expression levels [represented as log2 (RPKM means)]; red indicates high expression level, blue indicates low expression level, and white indicates RPKM = 0; **C** Expression analysis of differentially expressed genes in citric acid and glyoxylate cycle pathway of the Chinese fir roots under different phosphorus concentrations (high Pi supply - HP, Pi deficiency - LP and Pi starvation -NP) for 3 and 30 days. The experiments were repeated three times on three biological replicates of each treatment (*n* = 3). Values are means ± SD. Bars followed by different letter(s) indicate significant difference at *P* < 0.01

Citric acid and glyoxylate cycle play an important role in the synthesis of organic acids in plant tissues, and the transcriptome sequencing of Chinese fir roots under phosphorus starvation revealed that the main metabolic pathway for enrichment of differentially expressed genes was the biosynthetic pathway of organic acids. The annotated transcripts related to citric acid and glyoxylate cycle were mapped, and 28 genes with significant differences in enzyme coding presumably involved citric acid and glyoxylate cycle acid were identified under Pi starvation (Fig.5A). Differential gene expression thermograms showed that 11 genes were up-regulated, 12 genes were down-regulated and 5 genes had no specific expression pattern (Fig.5B). In general, the metabolism of pyruvic acid in Chinese fir roots was enhanced under Pi deficiency, and 5 pyruvic dehydrogenase genes were up-regulated to promote the synthesis of citric acid. At the same time, the expression of isocitratelyase Unigene0025798 in glyoxylic acid synthesis pathway was also increased to promote glyoxylic acid synthesis. The expression of malate synthase Unigene003021 was up-regulated to promote malic acid synthesis under Pi deficiency, therefore, the synthesis of citric acid, malic acid, glyoxylic acid and other organic acids could be promoted under Pi deficiency.

In addition, qRT-PCR analysis was used to study the expression of transcripts in citric acid and glyoxylate cycle pathway, including pyruvate dehydrogenase, citrate synthase, aconitase, isocitrate dehydrogenase and isocitrate lyase (Fig.5C). The results of expression verification were consistent with the main expression characteristics of heat map. As a whole, Unigene0051978, Unigene0080699, Unigene0007996 were up-expressed and Unigene0074595 were down-expressed with the enhancement of Pi deficiency and the extension of time. Thus, the up-regulation of pyruvate dehydrogenase promoted the synthesis of acetyl-CoA by pyruvate. Likewise, the up-regulation of citric acid synthase promoted the synthesis of citric acid, and the down regulation of aconitase reduced the decomposition of citric acid. The up-regulation of isocitrate dehydrogenase enhanced the synthesis and metabolism of organic acids in the root system of Chinese fir by promoting the synthesis of α-ketoglutaric acid.

### The contents of organic acids and the activity of related enzymes

**Fig. 6:**
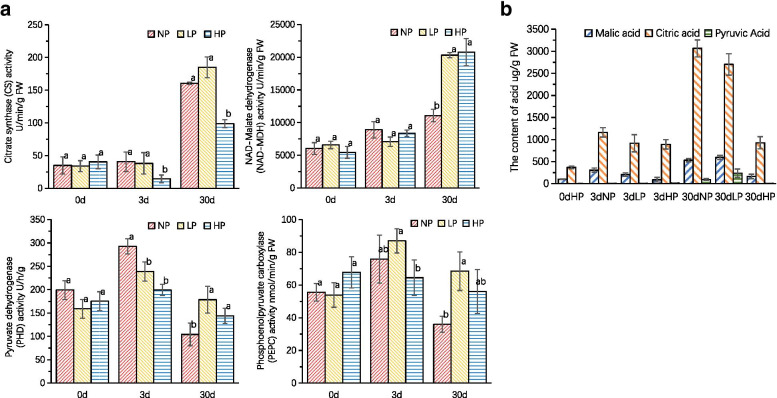
**A** Activities of citrate synthase, CS, NAD-malate dehydrogenase, NAD-MDH, pyruvate dehydrogenase, PDH, and phosphoenolpyruvate carboxylase, PEPC, in roots of Chinese fir seedlings exposed to different phosphorus treatments; **B** Contents of malic acid and citric acid in roots of Chinese fir seedlings treated with different phosphorus supply for 0, 3 and 30 days. Values are means ± SD (*n* = 3)

The activities of NAD-malate dehydrogenase, citrate synthase, pyruvate dehydrogenase and phosphoenolpyruvate carboxylase in the root of Chinese fir seedlings after 0, 3 and 30 days of different phosphorus treatments were determined (Fig. 6A). There was no significant difference in activity of NAD-malate dehydrogenase under different phosphorus treatments after 3 days. However, the activity of NAD-malate dehydrogenase under Pi deficiency condition after 30 days was significantly lower than that under normal Pi supply. Compared with the normal Pi supply, the activity of citric acid synthase increased under the Pi deficiency condition after 3 and 30 days. The activity of pyruvate dehydrogenase increased significantly under Pi deficiency after 3 days, and increased with the increase of stress intensity, but decreased under Pi deficiency after 30 days with no significant difference.

The contents of malic acid, citric acid and pyruvic acid in roots of Chinese fir seedlings were measured after 0, 3 and 30 days of different phosphorus treatments (Fig. 6B). The contents of malic acid in the roots of Chinese fir were higher in Pi deficient condition than that under normal Pi supply after 30 days, and the higher the stress intensity, the higher the contents of malic acid. The content of citric acid in the roots of Chinese fir seedlings increased significantly after 3 days without Pi supply than that of the roots grown under normal Pi supply. Under Pi deficiency, pyruvate content was lower than that of normal Pi supply after3 days, but increased after 30 days.

## Discussions

### Plant growth, root morphology and nutrient element content under pi deficiency

Our study showed that the morphological and physiological responses of plants to Pi deficiency become prominent around 30 days after Pi deficiency. Pi deficiency inhibited seedling height growth but promoted root elongation. The root-shoot ratio of Chinese fir seedlings under Pi deficiency condition was higher than that under normal phosphorus condition, which is similar with the general morphological characteristics of Chinese fir under Pi deficiency condition [54, 55, 58]. Under Pi deficiency condition for 30 days, the content of phosphorus in root system decreased significantly. The contents of root-shoot ratio of P, K, Mg and Cu increased, and the ratios of Ca, Fe and Mn decreased. This indicates that Pi deficiency altered the absorption and transport of other nutrient elements in Chinese fir. In order to ensure high phosphorus absorption and utilization efficiencies, some Chinese fir genotypes developed adaptive mechanisms via increasing the root growth and biomass distribution to increase the contact zone between the soil and the root [68]. Furthermore, Chinese fir roots can release organic acids, protons or acid phosphatase, which activate fixed phosphorus in the soil and increase the available phosphorus content in the soil environment [57]. Chinese fir can also maintain its growth by breaking down dead fine roots and releasing phosphorus. Chinese fir can also activate the change and transduction of chemical signals in vivo under the condition of Pi deficiency. By dissolving the root cortical cells, activating the phosphate stored in the cell wall, enhancing the activity of high affinity inorganic phosphorus transporter, and increasing the photosynthetic efficiency of leaves, the utilization efficiency of phosphorus in vivo can be increased [20, 35].

### Transcriptional characteristics and differentially expressed genes in Chinese fir roots under pi deficiency stress

The root transcriptional characteristics of Chinese firYang061 clones were studied after 3 and 30 days of NP-, LP- and HP- treatment, and a total of 126,413 Unigenes were obtained, of which 50,808 Unigenes were annotated in the database. The analysis of differential expression of Unigenes showed that the stronger the Pi deficiency stress was, the larger the differentially expressed genes were, particularly after 30 days than 3 days of Pi deficiency. GO annotation revealed that the distribution of cell and cell subclass catalytic activity, protein binding and transport activities were enriched to a much higher proportion of genes than other items in the same category. This result showed that the root system of Chinese fir seedlings responded to P stress by changing its cell components, adjusting its catalytic activity and adapting to the stress environment through various metabolic pathways. The results of KEGG enrichment analysis showed that the response genes could be divided into more than 30 subclasses and distributed in more than 120 pathways, which include redox processes, transmembrane transport, osmotic stress response, pyruvate metabolism, tricarboxylic acid cycle and acid phosphatase.

The genes obtained by trend analysis were annotated, and the up-regulated genes included transcription factors involved in the stress response-signaling pathway. It also included the tonoplast dicarboxylate transporter gene, which is involved in the organic acid transport through the tricarboxylic acid cycle pathway, and isocitric acid and isopropyl malate dehydrogenase, which are involved in oxidative decarboxylation [18, 37]. Pi deficiency transcription factors such as MYB play an important role in plant responses to biotic and abiotic stresses [15, 50]. Under low phosphorus conditions, *Arabidopsis thaliana WRKY45* regulates the high expression of *PHT1* in the roots. In other plants, MYB member PHR1 can positively regulate miR399, SPXs, PHT1, and the downstream genes PHO2 and PAPs [15, 23]. The oxidative decarboxylation of organic acid transporters in tricarboxylic acid cycle is an important process for the synthesis and secretion of organic acids [11]. Thus, the root system of Chinese fir under Pi deficiency can enhance the expression of high affinity phosphate carrier genes and organophosphate hydrolase genes, thereby promoting the synthesis and secretion of organic acids to increase available phosphorus through chelating.

Among the acid phosphatase ACP genes, purple acid phosphatase PAP gene and SPX domain protein screened from the transcriptome data, 17 PAP genes were differentially expressed, three ACP genes and three SPX domain proteins were induced by Pi deficiency. The expression of ACP and PAP genes increased with the intensity of Pi deficiency and the time of stress, indicating that Chinese fir has the physiological response of synthesizing acid phosphatase to activate the organic phosphorus in its rhizosphere under Pi deficiency. Most of the acid phosphatase was involved in the Pi transfer and recovery process [17, 52]. They can be classified as intracellular acid phosphatase and secretory acid phosphatase, depending on whether they are secreted outside the cell [66]. The intracellular acid phosphatase is expressed in various tissues, and mainly plays a role in Pi recovery and inter-tissue transport in plants, and the secretory acid phosphatase plays a role in the hydrolysis of external organic phosphorus into Pi [46, 52]. Current studies on *Arabidopsis thaliana* [50] and tomato [44] have shown that both acid phosphatase can be induced by Pi deficiency to increase their up-regulation. Acid phosphatase, a metal hydrolase, has been found to induce up-regulation of PAP under Pi deficiency in a variety of plants.

### Enhancement of organic acid synthesis in Chinese fir under pi deficiency

One of the typical characteristics of plant roots in response to Pi deficiency is to enhance the synthesis and secretion of organic acids in roots to activate insoluble phosphate in soil. Root transcriptome of Chinese fir in response to Pi deficiency showed that most of the 20 differentially expressed genes were up-regulated after 30-day of Pi deficiency compared to 3-day Pi deficiency stress. This might be related to the time lag in phosphorus activation by organic acid synthesis in Chinese fir roots. Furthermore, genes related to citrate-glyoxylate cycle were generally different. Under 3-day of HP-treatment, the expression level of except-enzyme genes was low, but under 30-day of Pi deficiency, 15 of the genes on the 26 TCA metabolic pathway were up-regulated, nine were down-regulated and two were not detected. The expression of eight Unigenes increased with the increase of Pi deficiency and the extension of time. Compared with 3-day of Pi deficiency, the expression of genes related to the tricarboxylic acid cycle pathway was more active under 30-day of Pi deficiency, with the increase of stress degree. Thus, Chinese fir activated the enhanced synthesis and secretion of organic acids in roots to promote its survival in low soil phosphorus environment.

The contents of malic acid and citric acid in the roots of Chinese fir under 3-day of Pi deficiency increased, while the contents of citric acid in the roots of Chinese fir under 30-day of Pi deficiency significantly increased. The results showed that citric acid was more sensitive to Pi deficiency than malic acid. Citrate synthase was up-regulated under Pi deficiency with increasing stress intensity. Aconitase was up-regulated under Pi deficiency, however, the expression level decreased with the increase of stress intensity. In addition, isocitrate dehydrogenase always showed a relatively high level of expression, which was up-regulated under 3-day of Pi deficiency treatment, but decreased under 30-day of Pi deficiency. Succinyl-coA synthase was highly expressed under 30-day of Pi deficiency, and its expression increased with the increase of stress intensity. Similarly, under 30-day of Pi deficiency, the expression of isocitrate lyase and malate synthase was increased while the expression of malate dehydrogenase-related genes was down-regulated compared with that under 3-day of Pi deficiency. The increase of organic acid synthesis and secretion in roots of plants under Pi deficiency has been widely confirmed, but the selection of organic acid secretion is different in different species. For instance, the amount of citric acid secretion in roots of rape and *Lupinus albus* is evidently increased under Pi deficiency, whereas the exudation of malic acid and oxalic acid increased in soybean root system [3, 29, 34]. Since the production and exudation of organic anions is a process that requires more carbon than other processes, citric acid secretion may be an important means of phosphorus uptake by Chinese fir roots during the late stage of phosphorus starvation.

The activity of pyruvate synthase was up-regulated under Pi deficiency condition. This may be due to the extraction of organic anions below the detection limit, or pyruvic acid itself as a metabolic intermediate, which is also the raw material for the synthesis of amino acids, the starting substrate of tricarboxylic acid cycle, and the final product of glycolysis. Previous studies have shown that biosynthesis and exudation of organic anions are associated with increased expression of genes encoding PEPC, NAD-MDH, CS and PDH. However, it should be noted that functional compensation exists in genes and proteins, and the expression of genes is not consistent with the increase of enzyme level, enzyme activity and organic acid production. In general, the metabolism of pyruvic acid of Chinese fir was enhanced under Pi deficiency in this experiment, so more pyruvic acid was used to synthesize various organic acids in tricarboxylic acid cycle. The gene for pyruvate dehydrogenase was up-regulated, the synthesis of citric acid was promoted, and the expression of isocitrate dehydrogenase related genes was up-regulated to promote α-ketoglutaric acid synthesis. The α-ketoglutaric acid can synthesize more glyoxylic acid, fumaric acid and malic acid in the presence of dehydrogenase. The expression of ATP-citrate lyase in the glyoxylic acid synthesis pathway is enhanced to promote the synthesis of glyoxylic acid, malate synthase was also up-regulated to promote the synthesis of malic acid under Pi deficiency, thus promoting the synthesis of citric acid, isocitrate, malic acid, glyoxylic acid and other organic acids under Pi deficiency. This result is also consistent with the synthesis and secretion of organic acids by Chinese fir roots under Pi deficiency [20]. The results showed that under Pi deficiency, the proton secretion of Chinese fir root increased, the activity of acid phosphatase in root increased, and the pH of rhizosphere decreased [27]. In addition, the organic acid content of Chinese fir roots under Pi deficiency increased by 325% compared with that under normal phosphorus supply, and the increased organic acids were mainly acid and tartaric acid [1].

## Conclusions

Chinese fir roots showed increased expression of phosphorus starvation related genes, citrate and malate synthesis genes, increased content of organic acids and enhanced activities of related enzymes under Pi deficiency. We obtained three SPX domain genes, 17 purple acid phosphatase genes, three acid phosphatase genes and 35 phosphate transporter genes by screening the genes related to phosphorus starvation response, which in turn increased rhizosphere Pi. We also found 20 genes related to the metabolism of organic acid synthesis and 28 genes related to the metabolism of citric acid and glyoxylic acid that were significantly expressed. The up-regulated expression of the phosphorus transporter gene, promotes the absorption and transport of Pi in roots. The contents of citric acid and malic acid in the roots of Chinese fir were also increased and the activities of malic acid and citric acid synthase were increased after 30 days of Pi deficiency, which may be related to the adaptation of Chinese fir to Pi deficiency. Screening phosphorus starvation response genes laid a foundation for further research, verify the functions of the alternative genes and explore the genetic variation of tree morphology after the establishment of genetic transformation system of Chinese fir in the future. It provides genetic resources and data support for exploring the mechanism of phosphorus uptake by Chinese fir and cultivating phosphorus efficient genotypes of Chinese fir. The findings on the relationship between Pi deficiency and organic acid metabolism of Chinese fir provides a new way to reveal the molecular mechanism of response of Chinese fir roots to Pi deficiency and the pathway of organic acid synthesis in Chinese fir roots.

## Data Availability

The transcriptome data analyzed in the current study were deposited to National Center for Biotechnology Information - Sequence Read Archive (https://www.ncbi.nlm.nih.gov/sra). Accession number: PRJNA681878.

## Abbreviations

*Cl*: *Cunninghamia lanceolata* (Lamb.) Hook
NP: Phosphorus starvation
LP: Low phosphorus supply
HP: High phosphorus supply
Pi: Available phosphorus
P: Phosphorus
Ca: Calcium
Fe: Ferrum
Al: Aluminium
O: Oxygen
K: Kalium
Mg: Magnesium
Cu: Cuprum
Zn: Zinc
Mn: Manganese
DW: Dry weight
RPKM: Reads per kilobase per million mapped reads
CDS: The coding sequence
NCBI SRA: National center for biotechnology information - Sequence Read Archive
Nr: NCBI non-redundant protein databases
GO: Gene Ontology
KOG: EuKaryotic ortholog groups
KEGG: Kyoto Encyclopedia of Genes and Genomes
TCA cycle: Tricarboxylic acid cycle
RNA: Ribonucleic Acid
ATP: Adenosine triphosphate
PHR1: Phosphate starvation gene
PHT: Phosphate transporter
PHO1: PHOSPHATE1
ABC: ATP Binding Cassette
SPX: SYG1/Pho81/XPR1
ACP: Acid phosphatase
PAP: Purple acid phosphatase
CS: Citrate synthase
NAD-MDH: NAD-malate dehydrogenase
PDH: Pyruvate dehydrogenase
PEPC: Phosphoenolpyruvate carboxylase
